# Sleepiness induced by sleep-debt enhanced amygdala activity for subliminal signals of fear

**DOI:** 10.1186/1471-2202-15-97

**Published:** 2014-08-19

**Authors:** Yuki Motomura, Shingo Kitamura, Kentaro Oba, Yuri Terasawa, Minori Enomoto, Yasuko Katayose, Akiko Hida, Yoshiya Moriguchi, Shigekazu Higuchi, Kazuo Mishima

**Affiliations:** Department of Psychophysiology, National Institute of Mental Health, National Center of Neurology and Psychiatry, 4-1-1 Ogawa-Higashi, Kodaira, Tokyo, 187-8553 Japan; Graduate School of Integrated Frontier Science, Kyushu University, 6-10-1 Hakozaki, Higashi-ku, Fukuoka, 812-8581 Japan; Research Fellow of the Japan Society for the Promotion of Science, 5-3-1, Kojimachi, Chiyoda-ku, Tokyo, 102-0082 Japan; Integrative Brain Imaging Center, National Center of Neurology and Psychiatry, 4-1-1 Ogawa-Higashi, Kodaira, Tokyo, 187-8553 Japan; Department of Psychology, Keio University, 4-1-1Hiyoshi, Kohoku-ku, Yokohama-shi, Kanagawa, 223-8521 Japan; Faculty of Design, Kyushu University, 4-9-1 Shiobaru, Minami-ku, Fukuoka, 815-8540 Japan

**Keywords:** Sleepiness, Nonconscious, Unconscious, Subliminal, Emotion, Fearful face, Amygdala

## Abstract

**Background:**

Emotional information is frequently processed below the level of consciousness, where subcortical regions of the brain are thought to play an important role. In the absence of conscious visual experience, patients with visual cortex damage discriminate the valence of emotional expression. Even in healthy individuals, a subliminal mechanism can be utilized to compensate for a functional decline in visual cognition of various causes such as strong sleepiness. In this study, sleep deprivation was simulated in healthy individuals to investigate functional alterations in the subliminal processing of emotional information caused by reduced conscious visual cognition and attention due to an increase in subjective sleepiness. Fourteen healthy adult men participated in a within-subject crossover study consisting of a 5-day session of sleep debt (SD, 4-h sleep) and a 5-day session of sleep control (SC, 8-h sleep). On the last day of each session, participants performed an emotional face-viewing task that included backward masking of nonconscious presentations during magnetic resonance scanning.

**Results:**

Finally, data from eleven participants who were unaware of nonconscious face presentations were analyzed. In fear contrasts, subjective sleepiness was significantly positively correlated with activity in the amygdala, ventromedial prefrontal cortex, hippocampus, and insular cortex, and was significantly negatively correlated with the secondary and tertiary visual areas and the fusiform face area. In fear-neutral contrasts, subjective sleepiness was significantly positively correlated with activity of the bilateral amygdala. Further, changes in subjective sleepiness (the difference between the SC and SD sessions) were correlated with both changes in amygdala activity and functional connectivity between the amygdala and superior colliculus in response to subliminal fearful faces.

**Conclusion:**

Sleepiness induced functional decline in the brain areas involved in conscious visual cognition of facial expressions, but also enhanced subliminal emotional processing via superior colliculus as represented by activity in the amygdala. These findings suggest that an evolutionally old and auxiliary subliminal hazard perception system is activated as a compensatory mechanism when conscious visual cognition is impaired. In addition, enhancement of subliminal emotional processing might cause involuntary emotional instability during sleep debt through changes in emotional response to or emotional evaluation of external stimuli.

## Background

Perceptual information that elicits emotional responses is partially processed without surfacing to the conscious mind, and the subcortical brain region is thought to play various roles in this process. Even a subliminal emotional stimulus can elicit a specific physiological response. For example, the amygdala, the brain area responsible for emotional cognition, is activated even when the subject is not aware of any emotional stimulus due to backward masking of a brief visual stimulus by a different emotional stimulus presented immediately following the target stimulus [1–3] or due to binocular rivalry [4, 5]. Subcortical regions such as the superior colliculus, pulvinar, and basal ganglia mediate the signal transduction pathway responsible for such physiological responses. Although it has been conventionally thought that signals were transmitted from the retina to the amygdala via the superior colliculus and pulvinar [2, 3, 6, 7], there is now some debate over the details following a recent proposal that the subcortical signal pathway has a complex structure comprising several branches [8].

Representative findings in support of the subliminal processing of emotions indicate that when presented with emotional facial expressions, patients with visual cortex damage discriminated emotional valence of facial expressions in the absence of conscious visual experience [9], showed different physiological responses for each emotion [10], and had an activated subcortical pathway on functional brain imaging [11]. Such phenomena were once thought to be special examples of an otherwise hidden mechanism in healthy individuals that is only exposed by a loss of conscious visual cognition. However, this subliminal process might be utilized in healthy individuals with temporally or functionally impaired visual cognition.

One of the factors in daily life that can affect visual cognition is sleep debt. Simulation studies of sleep debt (i.e., overnight total sleep deprivation) consistently show that, in addition to a marked decline in viewing-task performance, reduced activity in the parietal and occipital lobes involved in visual cognition and attention is proportional to individual vulnerability to sleep deprivation [12]. Similar results were obtained from cognitive tasks on working memory [13], vigilance [14], language learning [15], and logical thinking [16], indicating that a decline in task performance is caused by a functional decline in visual cognition and attention due to sleep debt.

Swann et al. reported shortened response times to subliminal priming after 2 days of short sleep, and stated that a decline in high-order supraliminal function due to sleepiness activated a compensatory subliminal function [17]. Furthermore, the subliminal process might work as a system that compensates for the decline seen in the level of consciousness with strong sleepiness, a situation that poses considerable danger to survival. For example, as a prey animal, a certain rabbit species sleeps with their eyes open in the wild, during which time their electroencephalograms show brain activation due to visual stimuli [18]. This is advantageous because it enables the animals to respond quickly to an attack by natural enemies, by waking up and engaging in avoidance behavior. Subliminal hazard perception might also be preserved in humans by activation via the pathway for subliminal information during episodes of strong sleepiness.

In this study, sleep debt was simulated to determine whether an increase in sleepiness induces a decline in conscious visual cognition and attention as well as activation of subliminal emotion processing.

## Methods

### Ethics

This study was approved by the Ethics Committee of the National Center of Neurology and Psychiatry, Japan, and was conducted in accordance with the Declaration of Helsinki.

### Participants

Participants were 14 healthy, right-handed adult men (mean ± standard deviation age, 24.1 ± 3.32 years) who provided written informed consent prior to participating in the study. The participants’ sleep schedule was monitored using a sleep log and actigraph (Ambulatory Monitoring Inc., Ardsley, NY) during a 2-week period prior to the study (observational period) and during the subsequent experimental period. Sleep-onset time, wake time, and the amount of time awake in bed were calculated from actigraph data according to Cole’s algorithm with optimal parameters [19], and were compared with the sleep log to confirm the absence of irregular life patterns, such as shiftwork and staying up all night. Overnight polysomnography was conducted to screen for sleep disorders during the observational period.

Exclusion criteria were as follows: mean bedtime or wakeup time during the observational period outside of the hours 23:00–02:00 and 07:00–10:00, respectively; some form of sleep disorder; serious physical complication; psychiatric disorder; ocular disease, including achromatopsia; taking medication or substances that might affect the experimental data (e.g. steroids and drugs that induce drowsiness such as hypnotics and antihistamines); having an implanted metal object such as a pacemaker; performing shiftwork; or having traveled within 3 months before the study to a country with more than a 6-hour time difference. This study also excluded individuals who consumed more than 200 mg of caffeine per day and heavy smokers who were unable to quit smoking for 5 days.

### Sleep restriction protocol

All participants attended a briefing session for the study, underwent sleep electroencephalography screening during the 2-week observational period, and participated in two 5-day experimental sessions. The number of hours in bed (lights out, permission to sleep) was 8 h/day in the sleep control (SC) session and 4 h/day in the sleep debt (SD) session. Both sessions were conducted as a crossover study with a 2-week interval between the sessions. To maintain a regular lifestyle during the interval, participants were restricted from staying up all night or performing shiftwork.

In the SC session, based on the sleep log and actigram from the observational period, mean bedtime (23:00–02:00) was used as the start time for 8 h of sleep (wakeup time 07:00–10:00). In the SD session, bedtime started 4 h later (03:00–06:00) than that in the SC session and total hours in bed were 4 h (wakeup time 07:00–10:00).

In both sessions, participants stayed home for the first 3 days and then stayed in a laboratory at the National Center of Neurology and Psychiatry for the next 2 days. To maintain a strict wakeup time at home, we sent an email alert every 4 h starting at the scheduled wakeup time until bedtime, and asked participants to answer the email immediately. In the laboratory, participants were under video camera surveillance, always assisted by a research attendant, and verbally awakened when in a drowsy state, such as when taking a nap or dozing off. During the wake period, participants were allowed to move freely around the laboratory, read and write, enjoy music and videos, play videogames, and engage in conversation with a researcher. Mineral water was always available, but caffeine and alcohol intake and heavy exercise were restricted. Ambient temperature and relative humidity in the laboratory were maintained at 25 ± 0.5°C and 50 ± 5%, respectively.

### MRI and emotional face-viewing task

**Figure 1:**
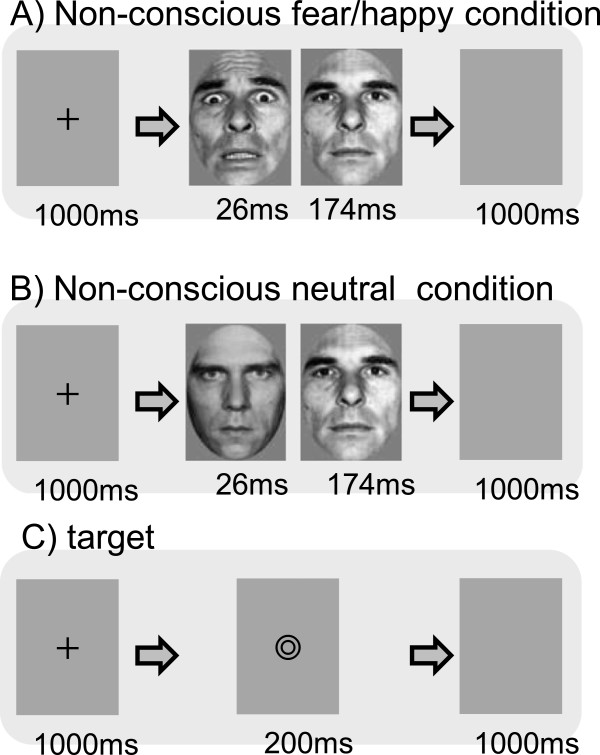
**Schematic illustration of emotional face-viewing tasks using backward masking. A)** In the nonconscious + fear task, a 26-ms presentation of a fearful face was followed by a 174-ms presentation of a neutral face of the same individual to mask the fearful face. **B)** In the nonconscious + neutral task, a 26-ms presentation of a neutral face was masked by a 174-ms presentation of a neutral face of another individual. **C)** Instead of a facial image, a specific symbol was randomly presented to maintain alertness, to which participants were asked to respond by pushing a button.

Magnetic resonance imaging (MRI) was performed on day 5 of each session. Participants were served the same breakfast (approximately 350-kcal sandwich) within 2 h of the wakeup time, completed a questionnaire about subjective sleepiness and mood in a room adjacent to the MRI room 2–2.5 h after the wakeup time, and underwent MRI 3–5 h after the wakeup time.During MRI, participants viewed emotional facial expressions presented under two different conditions: (1) the conscious condition, which provided sufficient viewing time to allow supraliminal visual perception of an emotional expression; and (2) the nonconscious condition, which provided a brief viewing time to allow for subliminal perception of an emotional expression followed by a neutral expression to mask the emotional facial image (Figure 1).

From the portraits of 16 individuals (8 men, 8 women) in two standardized image sets [20, 21], a total of 48 images of fearful, happy, and neutral facial expressions (16 individuals × 3 facial images per person) were selected and these images were presented after making the hair and background consistent.(1) Under the conscious condition, a fixation image was presented for 1000 ms followed by one of the three types of facial expressions for 200 ms and then a blank image for 1000 ms. (2) Under the nonconscious condition, after presenting a fixation image for 1000 ms, one of the three types of facial expressions was presented for 26 ms, followed immediately by a neutral facial image of another person of the same sex for 173 ms (backward masking), and then by a blank image for 1000 ms (Figure 1).

In both the conscious and nonconscious presentations, one trial consisted of presentations of a fixation image, a facial image, and a blank image. In both the conscious and nonconscious presentations, one trial consisted of presentations of a fixation image, a facial image, and a blank image. Each block consists of 9 trials of which 8 trials present either fearful, happy, or neutral facial images, and one trial shows a double circle symbol stimulus (target) used to keep participants alert and focused on the images, to which they responded by pressing a button. At the end of each block, a fixation image was shown on the screen for 15 s (baseline). A total of 12 image presentation blocks were conducted under conscious and nonconscious conditions (6 blocks each) in one session, and two sessions were performed with a 2-min break between the sessions. Each of six kinds of task block (conscious-fear, conscious-happy, conscious-neutral, nonconscious-fear, nonconscious-happy or nonconscious-neutral) was included 2 times in each session (4 times in total). The order of block presentation was counterbalanced between participants and sessions. The task program script was coded using Presentation software (Neurobehavioral Systems Inc., http://www.neurobs.com). The projector’s refresh rate was set to 75 Hz during task presentation (F22SX+, Projectiondesign Inc., Fredrikstad, Norway).

After completing each session, participants reported subjective sleepiness experienced during the face-viewing task and their awareness of the backward-masked image (presented for 26 ms) as follows: for subjective sleepiness, 0, not sleepy at all; 1, slightly sleepy; 2, sleepy; 3, very sleepy; and 4, slept a little; and for awareness of backward-masked images: 1, did not notice; 2, noticed something was presented but could not discriminate the faces; 3, could discriminate the faces; and 4, could see everything.

None of the participants responded with 4. Participants who responded with 3 were asked to describe the images verbally, and if they correctly described the facial images (i.e., fearful, happy, or neutral face of another individual), they were excluded from the analysis of the particular emotional category.

Three participants noticed a fearful expression image at least once, 7 participants noticed a happy expression, and one participant noticed a neutral expression (this participant also noticed a fearful face). Due to the small sample size, data for happy facial images were excluded from subsequent analyses. Eleven participants (mean ± standard deviation age, 24.5 ± 3.67 years) were unaware of the fearful or neutral facial images, and therefore only their data were analyzed.

### MRI acquisition

A Siemens Magnetom Verio 3 T MRI system was used to obtain MR images. To obtain reference images for analysis, structural images (T1-weighted magnetization-prepared rapid gradient-echo [MPRAGE] images) were taken with the following sequence parameters: TR/TE = 1900/2.52 ms, voxel size = 1× 1× 1 mm, flip angle 9°, and field of view = 256 × 192 mm.

To obtain task-related functional MRI (fMRI) images, single shot echo-planar imaging parameters were set at TR/TE = 2500/25 ms, 30 axial slices, voxel size = 3 × 3 × 4 mm, 1-mm interslice gap, flip angle 90°, matrix size = 64 × 64, and field of view = 192 × 192 mm. In each session, the first 5 of 137 scanning images were excluded from analysis.

### fMRI data analysis

Functional brain imaging data were analyzed using SPM8 (Wellcome Department of Imaging Neuroscience, http://www.fil.ion.ucl.ac.uk/spm/software/spm8/). For each image, motion and slice-timing correction as well as co-registration into an MPRAGE structural image was performed. The Montreal Neurological Institute (MNI) template was used for spatial normalization, and smoothing was performed using an 8-mm full width half maximum Gaussian kernel. MRI time-series data that contained three-dimensional blood-oxygenation-level-dependent (BOLD) signals of each participant were analyzed using the first-level fixed effects model with general linear model regression analysis. Using the canonical hemodynamic response function implemented in SPM8, a hypothetical hemodynamic time course corresponding to the stimulus presentations under each face-viewing task condition was developed by convolving the hemodynamic response function. Incorporated into the design matrix were 13 hemodynamic models of time series corresponding to the following: i) 6 conditions [3 categories of emotions (happy, fear, and neutral) × 2 types of image presentation (conscious and non-conscious)], ii) target image presentation, and iii) 6 head motions as regressors. Actual BOLD signals were analyzed voxel by voxel using the general linear model (GLM), and during presentation of either a fearful or neutral facial image the parameter estimate for each regressor was calculated and a beta image was generated. Significance was set at *p* < 0.001 (a cluster of > 5 voxels). Also, we took a region of interest (ROI) approach where we searched for significant clusters that survived multiple comparison correction with family-wise error (FWE) within the amygdala mask based on Anatomical Automatic Labeling (AAL) (*p* < 0.05, small volume correction [22]). The random-effects model was used to analyze between-subjects variability. A paired *t*-test was performed to calculate the *t*-value for the first *t*-level contrast value between each SC and SD session. Beta images for the presentation of nonconscious fearful, nonconscious neutral, and each fear-neutral contrast image were used in this analysis.

The *t*-test revealed no significant difference in amygdala activity during nonconscious face-viewing tasks between the SC and SD conditions (peak MNI coordinate, left amygdala: *x* = −14, *y* = −10, *z* = −18, *t*(10) = −1.93; right amygdala: *x* = 18, *y* = −8, *z* = −12, *t*(10) = −2.51).

### Correlation analysis with subjective sleepiness

To investigate whether sleepiness modulates implicit emotional processing, we correlated brain activity during presentation of fearful and neutral facial images with sleepiness during the task in SD and SC sessions. Significance was set at *p* < 0.001 (a cluster of > 5 voxels). We searched for significant clusters that survived multiple comparison correction with FWE within the amygdala mask based on AAL (*p* < 0.05, small volume correction [22]). Beta images for the presentation of nonconscious fearful, nonconscious neutral, and each fear-neutral contrast image were used in this analysis.

### Functional connectivity between the amygdala and superior colliculus

To determine whether the amygdala is functionally connected to remote regions and whether changes in this connectivity are related to changes in subjective sleepiness, we conducted functional connectivity analysis, seeded in the clusters in the bilateral amygdala that we obtained from results of the fear-neutral contrast. Because we were particularly interested in the subliminal visual pathway based on a previous study [1], we placed the target ROI in the superior colliculus as this region transmits visual information to the amygdala directly. Using WFU PickAtlas software from the SPM Toolbox, a mask for the superior colliculus was generated based on peak coordinates from results of the previous study.

Functional connectivity between the amygdala and superior colliculus (Fc_AMG-SCo_) was calculated using CONN toolbox version 13.1 (Alfonso Nieto-Castanon, http://www.alfnie.com/software/conn). Voxel-by-voxel GLM analyses were conducted, with the regression of time-series data in each voxel within the target ROI (superior colliculus) on the time-series data in the seed region. Head motions and hypothetical hemodynamic response to the main event (confounding effects of stimulus-locked transients [23]) were used as regressors. Bandpass-filter range was set at 0.008-0.09 Hz. Individual GLM analyses produced individual estimation maps (beta-maps) for the two sleep conditions (SD or SC) and the two kinds of facial presentation (nonconscious fearful or nonconscious neutral). We first checked the existence of statistically significant FcAMG-SCo during observation of fearful and neutral faces separately using t-tests, and also checked the differential FcAMG-SCo between fearful and neutral faces. Next, the individual beta maps in each facial condition were fed into the correlation analysis with the sleepiness at the time when the data were obtained.

Results were considered significant if *p* was less than 0.001 and the number of continuous voxels forming a cluster was greater than 5. Furthermore, we searched for significant clusters that survived multiple comparison correction with FWE within the superior colliculus mask (*p* < 0.05, small volume correction [22]).

### Increased sleepiness by sleep deprivation and changes in amygdala activity and functional connectivity to the superior colliculus

Next, we examined whether an increase in sleepiness by sleep deprivation promotes changes in amygdala activity and/or functional connectivity between the amygdala and superior colliculus (Fc_AMG-SCo_). We first calculated individual differential sleepiness between two different sleep states (SD vs. SC) and differential amygdala activation and differential Fc_AMG-SCo_ between the unconscious fear and neutral faces. We then evaluated the correlation of individual differential sleepiness with differential amygdala activation and differential Fc_AMG-SCo_. We searched for clusters in the bilateral amygdala whose activity (in response to fear vs. neutral faces) was correlated with sleepiness, and for clusters in the superior colliculus that showed significant functional connectivity to the amygdala during observation of fearful faces. MarsBar software (Matthew Brett, http://marsbar.sourceforge.net /marsbar.pdf) was used to calculate mean contrast values in each cluster.

### Statistics

SPSS PASW Statistics 18 software was used for statistical analysis. Behavioral indicators between SD and SC sessions were analyzed using the two-tailed *t*-test. Results are expressed as mean ± standard deviation. Between-subjects analysis was performed by calculating Pearson’s product moment correlation coefficient. Except for the analysis of functional brain activity, data were considered significant at *p* < 0.05.

## Results

### Sleep-time regulation

From the actigraph data, mean sleep time over the entire 5-day period in the SC and SD sessions was 8.13 ± 0.29 h (8 h 8 min ± 17 min) and 4.67 ± 0.56 h (4 h 40 min ± 34 min), with significantly fewer sleep hours (3.47 ± 0.61 h, or 3 h 28 min ± 37 min) in the SD session (*t*(10) = 19.00, *p* < 0.001).

### Subjective sleepiness

Subjective sleepiness scores for the SD session were significantly higher than those for the SC session (SC = 1.63 ± 0.84, SD = 2.5 ± 0.87, *t*(10) = 2.932, *p* < 0.05).

### Subjective awareness of masked images

No significant session-related differences were seen in either subjective awareness score (SC = 2.23 ± 0.52, SD = 1.95 ± 0.47, *t*(10) = 1.604).

### Button response

No significant session-related differences were seen in either the number or mean time of responses (SC = 2.23 ± 0.52, SD = 1.95 ± 0.47, *t*(10) = 1.604; SC = 11.63 ± 0.6, SD = 11.38 ± 1.16, *t*(10) = 0.71; SC = 596.98 ± 0.153.43, SD = 608.28 ± 115.34, *t*(10) = 0.11, respectively).

### fMRI data

**Figure 2:**
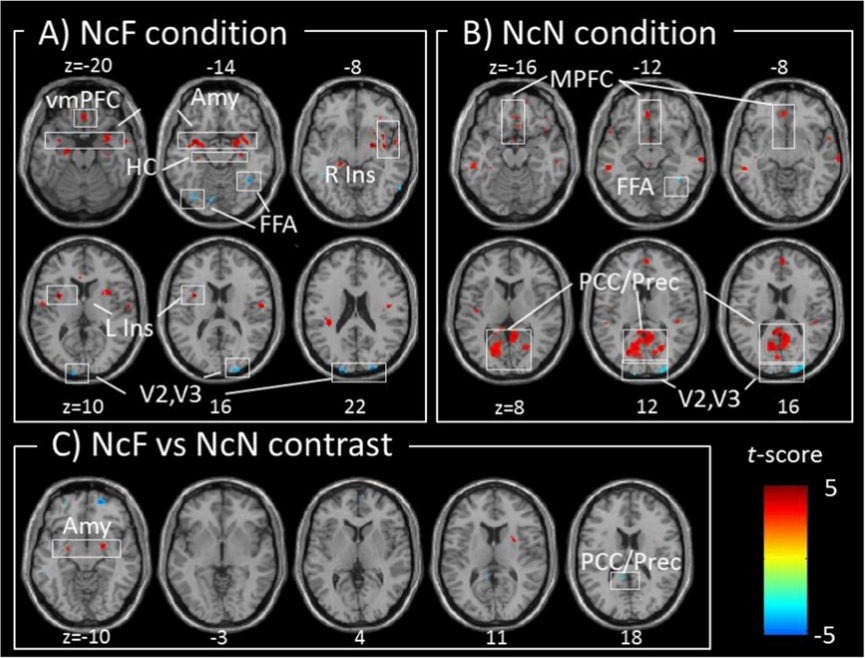
**Correlation analysis of the correlation with subjective sleepiness. A)–C)** Statistical parametric mapping of different brain regions showing a significant correlation with subjective sleepiness during a face-viewing task: **A)** nonconscious + fear contrasts, **B)** nonconscious + neutral contrasts, and **C)** nonconscious + fear vs. neutral contrasts. Red and blue areas show a significant positive and negative correlation with subjective sleepiness, respectively. **A)** In the nonconscious + fear contrasts, a significant positive correlation is observed in the bilateral amygdala, hippocampus, insula, and ventromedial prefrontal cortex, whereas a significant negative correlation is observed in the secondary and tertiary visual areas and the fusiform face area. **B)** In the nonconscious + neutral contrasts, a significant positive correlation is observed in the ventromedial prefrontal cortex, posterior cingulate cortex, and precuneus, whereas a significant negative correlation is observed in the secondary and tertiary visual areas and the fusiform face area. **C)** In the nonconscious + fear vs. neutral contrasts, the bilateral amygdala and posterior cingulate cortex show a significant positive and negative correlation, respectively. Abbreviations: NcF = Nonconscious fear condition; NcN = Nonconscious neutral condition; Amy = Amygdala; HC = Hippocampus; vMPFC = ventromedial prefrontal cortex; MPFC = medial prefrontal cortex; R Ins = Right Insula; L Ins = Left Insula; FFA = Fusiform face area; PCC = Posterior cingulate cortex; Prec = Precuneus; *p* < 0.001 uncorrected, cluster *k* > 5-voxel threshold; Degrees of freedom (*df*) = 20.

fMRI data are shown in Figure 2. Correlation analysis of fear contrasts showed a significant positive correlation between subjective sleepiness and activity in the amygdala, ventromedial prefrontal cortex, hippocampus, and insular cortex [peak MNI coordinates, left amygdala: *x* = −30, *y* = 0, *z* = −14, *t*(20) = 5.92, *p* < 0.05 small volume correction with FWE; right amygdala: *x* = 28, *y* = 0, *z* = −16, *t*(20) = 4.53, *p* < 0.05 small volume correction with FWE; ventromedial prefrontal cortex: *x* = 2, *y* = 34, *z* = −20, *t*(20) = 4.48; right hippocampus: *x* = 22, *y* = −14, *z* = −16, *t*(20) = 4.18; left hippocampus: *x* = −28, *y* = −22, *z* = −16, *t*(20) = 3.94; left insular cortex: *x* = −30, *y* = 4, *z* = 14, *t*(20) = 7.62; right insular cortex: *x* = 32, *y* = 8, *z* = 12, *t*(20) = 4.37 (Figure 2A)]. Also, as with the presentation of neutral facial images, subjective sleepiness was significantly negatively correlated with activity in the secondary and tertiary visual cortices and the fusiform face area (FFA) [left visual cortex: *x* = −16, *y* = −98, *z* = 24, *t*(20) = 4.61; right visual cortex: *x* = 24, *y* = −96, *z* = 22, *t*(20) = 5.13; left FFA: *x* = −40, *y* = −46, *z* = −10, *t*(20) = 4.41; right FFA: *x* = 40, *y* = −54, *z* = −12, *t*(20) = 5.15 (Figure 2A)].

On the other hand, correlation analysis of neutral contrasts revealed that subjective sleepiness was significantly positively correlated with activity in the default mode network (DMN) area (i.e., the precuneus, posterior cingulate cortex, inferior parietal gyrus, and medial prefrontal cortex [24, 25]) [precuneus: *x* = −4, *y* = −72, *z* = 28, *t*(20) = 6.03; left inferior parietal gyrus: *x* = −46, *y* = −48, *z* = 26, *t*(20) = 4.23; posterior cingulate cortex: *x* = −6, *y* = −24, *z* = 32, *t*(20) = 3.71; medial prefrontal cortex: *x* = 4, *y* = 56, *z* = 16, *t*(20) = 5.06 (Figure 2B)]. Furthermore, a significant negative correlation was observed between subjective sleepiness and activity in the secondary and tertiary visual cortices and the fusiform face area [left visual cortex: *x* = −10, *y* = −102, *z* = 18, *t*(20) = 4.21; right visual cortex: *x* = 24, *y* = −96, *z* = 22, *t*(20) = 5.95; right FFA: *x* = 38, *y* = −52, *z* = −10, *t*(20) = 3.80 (Figure 2B)].

**Figure 3:**
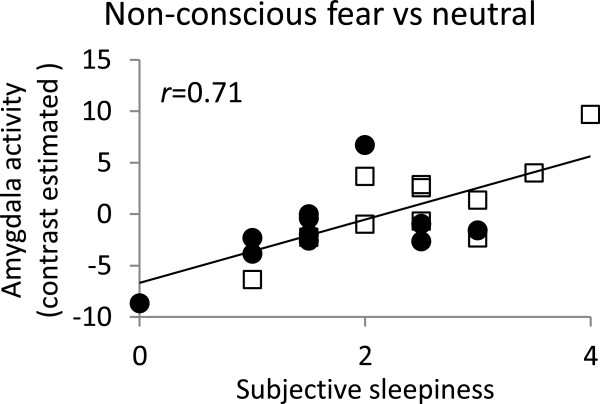
**Correlation scatter graph for subjective sleepiness and amygdala activity.** In fear-neutral contrasts, amygdala activity is significantly positively correlated with subjective sleepiness during a face-viewing task (*r*[20] = 0.71, *p* < 0.001). Amygdala activity was obtained by averaging clusters in the bilateral amygdala that showed a significant positive correlation in correlation analysis (*p* < 0.001, uncorrected). Data from the SC and SD sessions are shown in different colors in the graph. ●, SC; □, SD. *Abbreviations*: SC = sleep control condition; SD = sleep debt condition; Degrees of freedom (*df*) = 20.

Correlation analysis of fear-neutral contrasts showed a significant positive correlation between subjective sleepiness and activity in the bilateral amygdala [left amygdala: *x* = −24, *y* = −4, *z* = −8, *t*(20) = 5.05; right amygdala: *x* = 28, *y* = 8, *z* = −12, *t*(20) = 4.20 (Table 1, Figures 2C, 3), *p* < 0.05 small volume correction with FWE]. Activity in the precuneus, posterior cingulate cortex, and inferior parietal gyrus in the DMN area was significantly negatively correlated with subjective sleepiness [precuneus: *x* = −4, *y* = −74, *z* = 50, *t*(20) = 5.31; posterior cingulate cortex: *x* = −6, *y* = −24, *z* = 32 *t*(20) = 4.62; left inferior parietal gyrus: *x* = −40, *y* = −56, *z* = 54, *t*(20) = 5.31 (Table 1, Figure 2C)], presumably reflecting a positive correlation at the time of neutral-face presentation. Subjective sleepiness was not correlated with secondary or tertiary visual areas or the fusiform face area.

**Table 1:** **Coordinates of brain regions showing a significant correlation with subjective sleepiness in the correlation analysis of fear-neutral contrasts (** ***p*** **< 0.001, uncorrected,** ***k*** **> 5)**

Positive correlation									
	Brain region			BA		MNI		***t***	Cluster***k***
					x	y	z		
	Right	Lentiform nucleus/Parahippocampal gyrus	Putamen/Amygdala		24	−4	−8	5.05	25
	Right	Lentiform nucleus	Putamen		28	4	12	4.9	16
	Left	Parahippocampal gyrus	Amygdala		−24	−8	−12	4.2	17
**Negative correlation**									
	Left	Inferior parietal lobule		40	−40	−56	54	5.31	45
	Left	Precuneus		7	−4	−74	50	5.31	219
	Right	Superior frontal gyrus		10	22	56	−8	5.18	59
	Left	Fusiform gyrus		20	−40	−32	−22	4.83	26
	Right	Inferior temporal gyrus		20	34	−8	−42	4.78	33
	Left	Cingulate gyrus		23	−6	−24	32	4.62	74
	Left	Cerebellar tonsil			−50	−56	−42	4.22	6
	Left	Paracentral lobule		5	0	−44	62	4.22	55
	Left	Middle temporal gyrus		20	−56	−38	−12	4.09	5
	Right	Precuneus		7	18	−52	52	4.03	8
	Left	Superior frontal gyrus		11	−4	60	−22	4.02	11
	Right	Medial frontal gyrus		10	8	64	2	3.97	7
	Left	Posterior cingulate		29	−8	−44	16	3.96	11
	Left	Middle frontal gyrus		6	−36	−2	46	3.95	10
	Left	Middle frontal gyrus		11	−26	52	−14	3.82	14

The *x*, *y*, and *z* coordinates denote the peak location on the MNI template.

*Abbreviations*: BA = Brodmann area; MNI = Montreal Neurological Institute template; Cluster *k* = *p* < 0.001 uncorrected threshold; Degrees of freedom (*df*) = 20.

### Functional connectivity between the amygdala and superior colliculus

**Figure 4:**
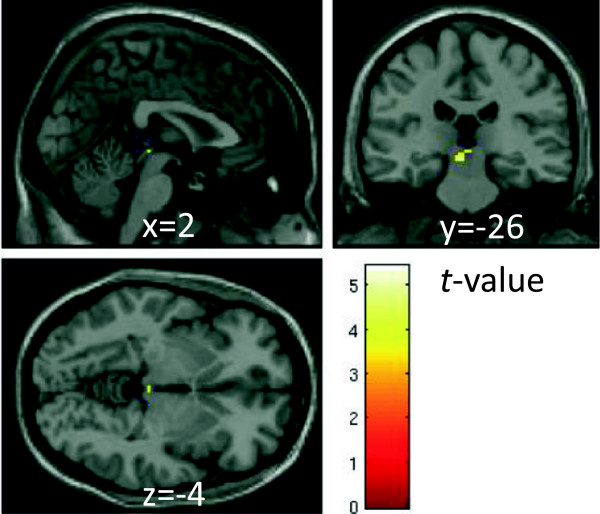
**Functional connectivity with the amygdala in fear contrasts.** The map shows significant functional connectivity between the bilateral amygdala and other voxels in the brain in both the SC and SD sessions. Significant connectivity was found in the cluster including the superior colliculus, peak MNI coordinate (*x*, *y*, *z*) = (−4, −26, −10) mm, *t*(21) = 5.43, *p* = 0.0001, *k* = 26 contiguous voxels. A significant cluster with a significant connection to the amygdala is rendered on a T1 anatomical referential image displayed by neurological convention, with the left side corresponding to the left hemisphere. MNI, Montreal Neurological Institute template.

We found a significant positive Fc_AMG-SCo_ during the presentation of fearful faces [peak MNI coordinates (mm) in the superior colliculus: *x* = −4, *y* = −26, *z* = −16, *t*(21) = 5.43, *p* < 0.05 small volume correction with FWE (Figure 4)]. We also found marginally significant Fc_AMG-SCo_ in the fear-neutral contrast [*x* = −4, *y* = −28, *z* = −8, *t*(21) = 3.45, *p* = 0.001]. On the other hand, no significant connectivity was found during observation of neutral faces [peak MNI coordinates: *x* = −4, *y* = −26, *z* = −10, *t*(21) = 1.75].

We did not find significant a correlation between subjective sleepiness and Fc_AMG-SCo_ for any type of stimulus [fear: *x* = −4, *y* = −32, *z* = −8, *t*(21) = 1.79; neutral: *x* = 0, *y* = −28, *z* = −10, *t*(21) = 2.76; fear vs. neutral: *x* = −6, *y* = −22, *z* = 0, *t*(21) = 2.66].

### Increased sleepiness by sleep deprivation and changes in amygdala activity and functional connectivity to the superior colliculus

**Figure 5:**
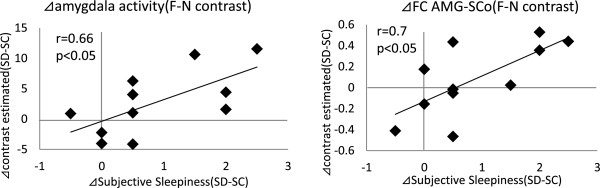
**Correlation between intersession differences in amygdala activity, amygdala–superior colliculus functional connectivity, and intersession differences in subjective sleepiness scores.** Intersession differences between sleep control and sleep debt sessions of amygdala activity and amygdala-superior colliculus functional connectivity in fear-neutral contrasts correlated positively with intersession differences in subjective scores, *r*(10) = 0.66, *p* < 0.05, *r*(10) = 0.70, *p* < 0.05, respectively. Δvalue, intersession difference between sleep control and sleep debt sessions for each value; SC, sleep control condition; SD, sleep debt condition; Fc_AMG-SCo_, functional connectivity between amygdala and superior colliculus.

After we calculated individual differential amygdala activation, differential Fc_AMG-SCo_, and differential subjective sleepiness between SD and SC, we obtained cross- correlations between differential amygdala activation, differential Fc_AMG-SCo_, and differential subjective sleepiness (Figure 5). Differential indices signify changes due to sleep deprivation (SD) compared to the SC session. Changes in amygdala activation and changes in Fc_AMG-SCo_ by SD were positively correlated with changes in the subjective sleepiness score [*r*(10) = 0.66, *p* < 0.05, *r*(10) = 0.70, *p* < 0.05, respectively (Figure 5)].

## Discussion

The results of this study revealed that the subjective feeling of strong sleepiness significantly altered participants’ emotional brain reaction toward negative emotional stimuli presented under nonconscious conditions. In particular, the intensity of amygdala activity in response to a fearful expression was significantly positively correlated with subjective sleepiness. On the other hand, in the secondary and tertiary visual areas and the fusiform face area specialized for visual cognition of facial expression [26, 27], activity was negatively correlated with subjective sleepiness. We found functional connectivity between the amygdala and superior colliculus (Fc_AMG-SCo_) during presentation of the fearful face. Further, changes in subjective sleepiness (the difference between the SC and SD sessions) were correlated with both changes in amygdala activity and Fc_AMG-SCo_ in response to subliminal fearful faces. These findings suggest that an increase in sleepiness enhances subliminal emotion processing that engages the amygdala and its connection to the superior colliculus. They also suggest that an increase in sleepiness enhances amygdala activity and overall subliminal emotion processing via the superior colliculus while at the same time reducing activity in areas involved in visual cognition.

The amygdala is known to play an important role in evoking negative emotional responses [28, 29]. Although visual perception of fearful facial expressions leads to amygdala activation in healthy individuals [30], this activation is more pronounced in patients with depression and anxiety disorder [31, 32]. On the other hand, when fearful expressions are presented nonconsciously or outside the focus of attention, amygdala activation is similar to that observed under conscious presentation [4, 5, 33].

The present findings revealed that activation of the amygdala in response to subliminal negative emotional stimuli was augmented by an increase in sleepiness. The amygdala is neurally connected to brain regions such as the insular cortex, medial prefrontal cortex, and hippocampus, and together they form the brain’s emotion network [34–36]. We found that activity of the emotion network in response to a fearful facial expression increased as sleepiness intensified. However, after subtracting the effect of the neutral expression, the amygdala was the only brain region significantly correlated with sleepiness. In other words, during strong sleepiness, the response to negative emotional stimuli was altered most noticeably in the amygdala.

During neutral facial image presentation, subjective sleepiness was significantly positively correlated with activity in the precuneus, posterior cingulate cortex, and medial prefrontal cortex. These regions form the DMN area, which is activated strongly during rest not involving tasks [24, 25]. Sleep deprivation was found to enhance activity in the DMN area during a task [14]. In the present study, sleep debt augmented activity in the DMN area during nonconscious presentation of neutral, but not fearful, expressions. Images that remained at the conscious level were neutral facial images under both presentations, suggesting that nonconscious presentation of fearful expressions might enhance attention toward subliminal warnings and prevent the default mode of activity in the DMN area.

Activity in the amygdala increased in participants who experienced intense sleepiness despite reduced responsiveness in the visual area involved in fearful expression. This suggests that subliminal visual signals are transmitted to the amygdala via an alternative pathway that bypasses the visual cortex [3, 5, 6, 11]. In the backward-masking task, a fearful expression was presented for a mere 26 ms without eliciting conscious perception, followed by presentation of a neutral expression long enough (176 ms) to ensure visual cognition. Under mild sleepiness (i.e., highly alert with functional attentional mechanisms), information on neutral facial expression is transmitted to the amygdala via the pathway involved in conscious vision, which might cancel out information from fearful (and fear-neutral) expressions transmitted via the subliminal pathway. When subjects were asked to perform an affect-labeling task to describe the emotion presented in the individual facial stimuli, self-reported distress toward the facial stimuli was reduced compared with the normal viewing task [37], and the activity of amygdala was suppressed [38, 39]. Given that the activity in the cortical area involved in visual face recognition was reduced under strong sleepiness in the present study, it is possible that affect labeling of currently presented faces (which remains at the conscious level) is impossible when highly sleepy, exposing the presence and impact of fearful information transmitted via the subliminal pathway.

In this study, nonconscious presentation of fearful facial expressions enhanced the activity of amygdala despite the reduced activity in the cortical area involved in facial recognition. The functional significance of this phenomenon might be that a primitive subliminal hazard perception system was utilized as an alternative mechanism in response to the functional decline in conscious emotion processing. The superior colliculus, which is present in the brain area presumably involved in the subliminal pathway, functions as the center of visual processing in fish and amphibians. On the other hand, the superior colliculus constitutes a small portion of the whole brain in humans, and therefore its function is considered phylogenetically old [40]. In ancient times, fearful facial expressions were important tools for conveying imminent life-threatening danger among tribal members and functioned as basic warning signs among them [41]. It is therefore possible that declines in supraliminal visual cognitive and attentional function due to strong sleepiness evoked strong reactions toward fearful expressions via the phylogenetically old subliminal pathway of emotional processing. It is also possible that the subliminal pathway is involved in the rapid transmission of danger-related information because subliminal information is reportedly transmitted faster than supraliminal information [10]. Furthermore, as shown in this study, activation of subliminal emotional processing due to enhanced sleepiness might also affect emotional adjustment during sleep debt. Although mood decline is likely to occur in response to mild stressors during sleep debt [42], sleep deprivation was reported to increase sensitivity to pleasurable experiences by lowering the threshold value in a task judging valence of pleasure-evoking pictures [43]. A change in nonconscious emotion processing can influence one’s conscious state, such as mood, feeling or emotional evaluation [44, 45]. Previous studies using nonconscious emotional stimuli have indicated that subjects can be unaware of their own emotional responses [11, 46]. According to the somatic marker hypothesis (see [47, 48] for details), changes in bodily response such as sympathetic hyperactivity can strongly affect subjective feelings and emotional evaluation without one’s conscious awareness of the responses. The amygdala and insula, where activation due to sleepiness was observed in this study, are involved in autonomic nervous system regulation [49, 50]. Our findings suggest that the enhancement of subliminal emotional processing during sleep debt might result in involuntary emotional instability, either directly or indirectly (e.g. through change of bodily response), without self-awareness. Because we did not measure sympathetic nervous system indices such as the respiratory and cardiac cycle in this study, we could not confirm that sympathetic activity accompanied enhanced amygdala activity during strong sleepiness. Moreover, the amygdala activation could be explained as the result of increased sympathetic activity due to sleep deprivation. Future studies should ideally measure indices such as heart rate and respiratory rate in the MRI scanner. In this study, however, the activity of the amygdala was changed by sleepiness only when observing fearful faces, not neutral faces, so a change in the sympathetic system does not completely explain the changes seen in nonconscious emotion responses, although sleepiness should impact subliminal emotional processing and alter amygdala activation.

## Conclusion

Sleepiness induced functional decline in brain areas involved in conscious visual cognition of facial expressions, but also enhanced subliminal emotional processing as represented by activity in the amygdala. These findings suggest that an evolutionally old and auxiliary subliminal hazard perception system is activated as a compensatory mechanism when conscious visual cognition is impaired. In addition, enhancement of subliminal emotional processing might cause involuntary emotional instability during sleep debt through changes in emotional response to or evaluation of external stimuli.

## Abbreviations

SD: Sleep debt
SC: Sleep control
MRI: Magnetic resonance imaging
fMRI: Functional MRI
MNI: Montreal neurological institute
BOLD: Blood oxygenation level dependent
GLM: General linear model
DMN: Default mode network.
